# Flow-through isolation of human first trimester umbilical cord endothelial cells

**DOI:** 10.1007/s00418-021-02007-7

**Published:** 2021-06-24

**Authors:** Michael Gruber, Elisa Weiss, Monika Siwetz, Ursula Hiden, Martin Gauster

**Affiliations:** 1 Division of Cell Biology, Histology and Embryology, Gottfried Schatz Research Center for Cell Signaling, Metabolism and Aging, Medical University of Graz, Neue Stiftingtalstraße 6, 8010 Graz, Austria; 2 Department of Obstetrics and Gynecology, Medical University of Graz, Auenbruggerplatz 14, 8036 Graz, Austria

**Keywords:** First trimester, Umbilical cord, Human placenta, Endothelial cells, Isolation, HUVEC, HUAEC

## Abstract

Human umbilical vein and artery endothelial cells (HUVEC; HUAEC), placental endothelial cells (fpAEC), and endothelial colony-forming cells (ECFC) from cord blood are a widely used model for researching placental vascular development, fetal and placental endothelial function, and the effect of adverse conditions in pregnancy thereon. However, placental vascular development and angiogenesis start in the first weeks of gestation, and adverse conditions in pregnancy may also affect endothelial function before term, suggesting that endothelial cells from early pregnancy may respond differently. Thus, we established a novel, gentle flow-through method to isolate pure human umbilical endothelial cells from first trimester (FTUEC). FTUEC were characterized and their phenotype was compared to the umbilical endothelium in situ as well as to other fetal endothelial cell models from term of gestation, i.e. HUVEC, fpAEC, ECFC. FTUEC possess a CD34-positive, juvenile endothelial phenotype, and can be expanded and passaged. We regard FTUEC as a valuable tool to study developmental processes as well as the effect of adverse insults in pregnancy in vitro.

**Supplementary Information:**

The online version contains supplementary material available at 10.1007/s00418-021-02007-7.

## Introduction

The endothelium is a versatile, multifunctional structure that continuously lines all blood vessels of the body. The formation of new blood vessels, the growth of existing blood vessels, and the transport of substances within the vessels and through the endothelium into the tissue are essential processes for the development and function of virtually all organs. Feto-placental circulation has formed by 6 weeks post-conception, which is followed by a dramatic increase in vascular growth and angiogenesis. Adequate endothelial growth and function crucially determine fetal development (Kaufmann et al. 2004; Mayhew et al. 2004). Various well-known metabolic, inflammatory, and environmental influences affect and disturb endothelial function in adults. These include, for instance, hyperglycemia and pro-inflammatory conditions (Prieto et al. 2014). Similarly, or even more, fetal endothelial function may be sensitive to disruptive insults during pregnancy. Pregnancy pathologies such as maternal gestational diabetes and pre-eclampsia also induce a hyperglycemic and/or pro-inflammatory environment (Carpenter 2007; Cvitic et al. 2014; Guillemette et al. 2015), and their effect on fetal endothelial function has been comprehensively researched (McElwain et al. 2020). Classically, research on normal and disturbed placental and fetal endothelial function uses human umbilical vein endothelial cells (HUVEC) or human umbilical artery endothelial cells (HUAEC), primary endothelial cells (EC) isolated from the large umbilical cord vessels after delivery. Both grow well in culture and can be passaged and expanded. HUVEC and HUAEC have been used to identify potential regulators of placental vascular development in normal pregnancy (Nagamatsu et al. 2004; Ma et al. 2021) and were employed to study the impact of adverse conditions, such as pregnancy pathologies, on endothelial dysfunction in vitro (Ying et al. 2021; Zheng et al. 2020; Saez et al. 2018). Also, the effect of viral infections on fetal endothelium was investigated using HUVEC in vitro (Richard et al. 2017).

In addition to umbilical cord EC, other, less common cell models have been employed to investigate fetal and placental endothelial function, including arterial and venous feto-placental EC derived from term chorionic plate arteries and veins (Lang et al. 2008). Moreover, endothelial progenitor cells have been isolated from cord blood and characterized as endothelial colony-forming cells (ECFC) (Ingram et al. 2008). However, all these cells represent fetal endothelial cell types obtained at the end of gestation, when endothelial growth and differentiation are basically complete. A fetal endothelial cell model for early pregnancy would enable and facilitate research into developmental processes of the feto-placental vasculature. Also, the effects of adverse conditions on endothelial function during early pregnancy may clearly differ from effects at the end of pregnancy. Therefore, we have established a novel method to isolate primary first trimester EC from umbilical cord vessels. The isolated primary cells were characterized with respect to their proliferation potential and phenotype and compared to other, common cell models for the feto-placental endothelium.

## Materials and methods

### Human placenta and cord blood samples

The study was approved by the ethical committee of the Medical University of Graz (31–019 ex 18-19 and 29-319 ex 16/17). First trimester placental tissue was obtained between weeks 6 and 12 of gestation with written informed consent from women undergoing legal elective surgical pregnancy termination. Term placental tissue and cord blood were obtained with written informed consent after cesarean section between 39 and 41 weeks of gestation.

### Isolation and culture of endothelial cells from first trimester umbilical cord vessel

**Fig. 1:**
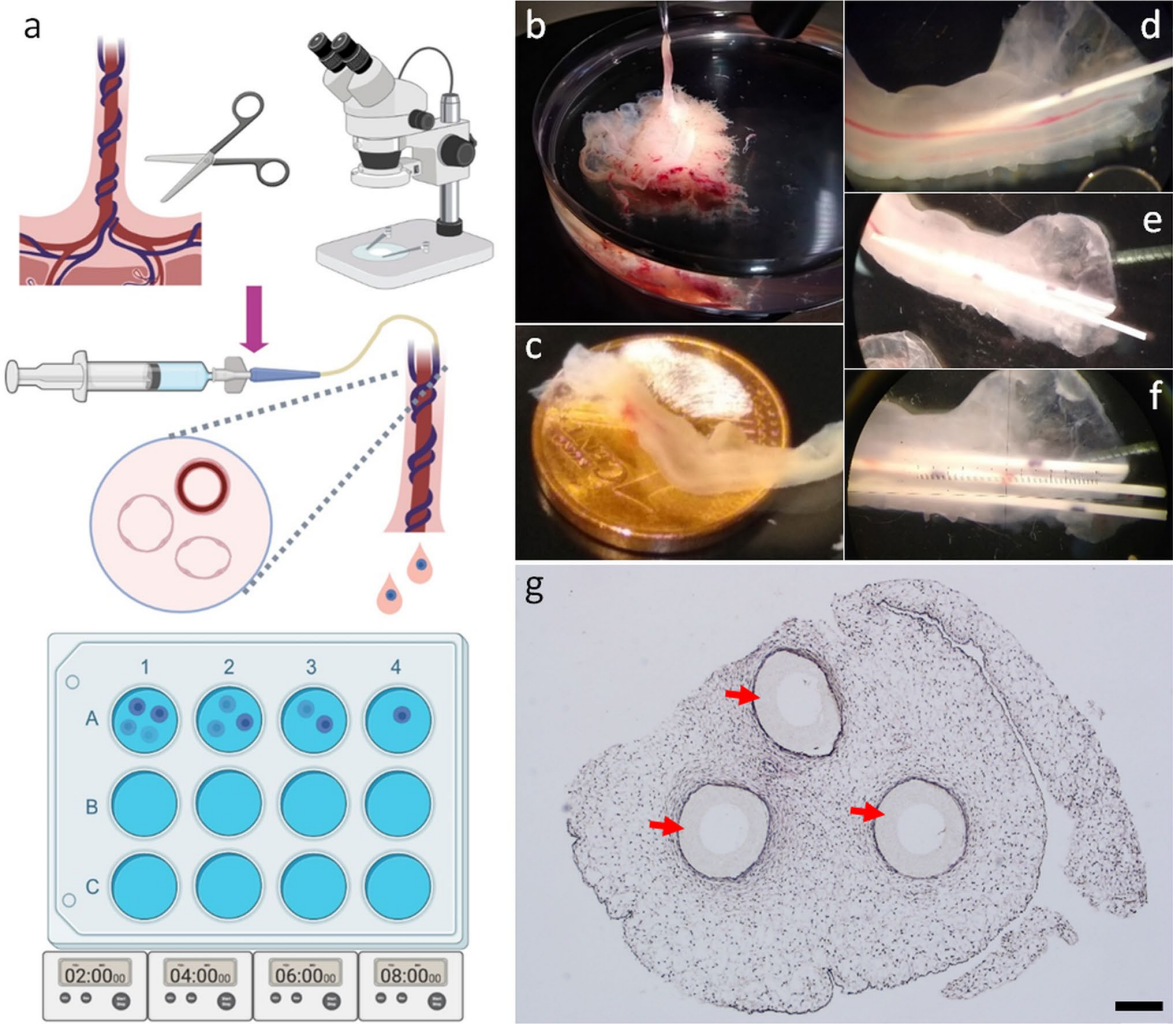
Human first trimester umbilical cord cannulation and isolation of endothelial cells. The isolation protocol included separation of the umbilical cord close to the cord insertion, followed by cannulation of the umbilical cord vessels under a stereomicroscope. Enzymatic flow-through digestion was followed by fractionated collection of umbilical cord EC (**a**). First trimester placenta tissue with umbilical cord (**b**), and size comparison with a 1 euro cent coin (**c**). Sequential cannulation of umbilical cord vessels using neonatal umbilical catheters (**d**–**f**). Successful cannulation was confirmed by HE staining of formalin-fixed paraffin-embedded (FFPE) first trimester umbilical cord sections, showing positioning of the catheters (red arrows) inside the cord vessels (**g**). Scale bar represents 200 µm. Figure parts were created with BioRender.com

Isolation of first trimester umbilical cord endothelial cells (FTUEC) was performed following a protocol previously described for chorionic plate-derived EC (Lang et al. 2008), with slight modifications. Umbilical cord segments of at least 2 cm length were dissected from placental chorionic tissue (Fig. 1a). The ends of the umbilical cord were cut off with a scalpel to generate straight vascular endings. Subsequently, umbilical cord vessels were cannulated with neonatal umbilical catheters (premicath, Ø 1 Fr., Vygon, Aachen, Germany) under a stereomicroscope. Microanatomical identification of cannulated vessels after fixation and embedding was achieved by staining the respective catheter beforehand with Mark-It Tissue Green Marking Dye (Thermo Fisher Scientific, MA, USA). Each vessel was rinsed with 3 ml of 1× Hanks’ Balanced Salt Solution (HBSS, Thermo Fisher Scientific). Then, the vessel was perfused with 2 ml of pre-warmed 0.5 mg/ml Collagenase/Dispase solution (Roche, Mannheim, Germany) diluted in HBSS and supplemented with 1% penicillin/streptomycin (P/S, GE Healthcare, IL, USA) for 8 min (flow rate 0.25 ml/min). The released cells were collected in four wells of a 12-well culture plate (Corning, NY, USA) pre-coated with gelatin (1% porcine skin gelatin, Sigma-Aldrich, MO, USA) by changing the collecting well every 2 min, generating four fractions (0.5 ml per well). Each well was pre-filled with 1 ml of culture medium (Endothelial Cell Growth Medium MV Kit, PromoCell, Heidelberg, Germany, supplemented with 0.1% gentamicin (Thermo Fisher Scientific), topped up to 10% fetal calf serum (FCS, HyClone, GE Healthcare) until the first medium change). Then, cells were cultured in ambient air at 5% CO_2_ and 37 °C in a humidified incubator. The next day, half of the culture medium was changed, followed by further complete media exchange every other day. After 1 week, the O_2_ concentration was set to 12%. Cells reached confluence on day 10 after isolation and were passaged. For that purpose, cells were washed with HBSS and incubated with 100 μl/well TrypLE™ Select cell-dissociation enzyme (Thermo Fisher Scientific) for 3 min at 37 °C. The cells of the four wells were pooled in 8 ml of culture medium and transferred to a 25 cm^2^ culture flask (Nunclon™, Thermo Fisher Scientific). The culture medium was changed twice a week and cells further expanded after reaching confluence.

For freezing, detached cells were resuspended in Dulbecco's modified Eagle medium (DMEM, Thermo Fisher Scientific) supplemented with 10% FCS and 1% P/S (‘DMEM complete’) and centrifuged at 800 rpm for 4 min at 4 °C. The cell pellet was resuspended (1.5 × 10^6^ cells/ml) in freezing medium consisting of culture medium containing 20% FCS and 10% dimethyl sulfoxide (DMSO, Sigma-Aldrich), and 1 ml of the suspension per cryo-vial (Greiner Bio-One, Kremsmünster, Austria) was frozen at −80 °C using a CoolCell^®^ (BioCision, CA, USA) in order to achieve a controlled low cooling rate of −1 °C/min. After 24 h, vials were transferred into liquid nitrogen for long-term storage. For thawing, the cryo vials were gently thawed using the ThawSTAR^®^ Cell Thawing System (MedCision, CA, USA) to ensure controlled temperature gradient conditions. The cell suspension was transferred to a 50 ml tube (Corning) containing 10 ml DMEM complete. After centrifugation at 800 rpm for 4 min, the pellet was resuspended in culture medium and seeded into a 75 cm^2^ culture flask. Media change was performed on the next day and cells were further cultured as described above.

### Isolation and culture of fpAEC and ECFC from human term placenta

Placental endothelial cells (fpAEC) and ECFC were isolated as described previously (Leopold et al. 2019). In brief, fpAEC were isolated from a chorionic plate artery via enzymatic digestion using Collagenase/Dispase, and incubated at 12% O_2_, 5% CO_2_ at 37 °C in a humidified incubator. ECFC were isolated from venous umbilical cord blood via density gradient centrifugation and incubated at 21% O_2_, 5% CO_2_ at 37 °C in a humidified incubator. After first passaging, O_2_ concentration was set to 12%.

### Isolation and culture of HUVEC from term placenta

For the isolation of term HUVEC, a washed 10 cm piece of cord was sealed on one end and filled with 10 ml of pre-warmed 0.5 mg/ml Collagenase/Dispase solution. After incubation for 8 min, the digested cells were collected in a tube containing 10 ml FCS and centrifuged for 7 min at 900 rpm. The cell pellet was resuspended in 10 ml culture medium supplemented with additional 5% FCS and transferred to a 75 cm^2^ culture flask pre-coated with gelatin. Cells were cultured at ambient air and 5% CO_2_ at 37 °C in a humidified incubator. Culture medium was changed partially on the next day and then twice a week. After 5 days of culture, the O_2_ concentration was set to 12%. After reaching confluence on day 7 after isolation, cells were expanded.

### Immunostaining of first trimester umbilical cord tissue and FTUEC

Human formalin-fixed paraffin-embedded (FFPE) first trimester umbilical cords (*n* = 5) were cut (5 μm) and mounted on Superfrost Plus slides (Thermo Fisher Scientific). After deparaffinization, slides were subjected to antigen retrieval by boiling in a microwave oven in 10 mM sodium citrate buffer (Merck, Darmstadt, Germany) pH 6.0 or 10 mM Tris-EDTA buffer (Merck) pH 9.0 (for 40 min at 150 W) or with pepsin (Sigma-Aldrich) for 30 min at 37 °C. After cooling for 20 min at room temperature, slides were transferred to TBS including 0.05% Tween 20 (TBS/T, Merck). To quench endogenous peroxidase, slides were incubated with UltraVision hydrogen peroxide block (Thermo Fisher Scientific) for 10 min, and after washing, nonspecific background was blocked by incubation with UltraVision Protein Block (Thermo Fisher Scientific) for 7 min. Primary antibodies (Table 1) were diluted in antibody diluent (Agilent, Dako, CA, USA) and incubated for 45 min at room temperature. After washing steps, the UltraVision Detection System HRP Polymer kit (Thermo Fisher Scientific) was used according to the user instructions. The polymer complex was visualized by incubating the slides with the AEC substrate kit (Abcam, Cambridge, UK) for 10 min. Following washing steps with distilled water, tissue sections and cells were counterstained with self-prepared Mayer's hematoxylin (Merck) and mounted with Kaiser’s glycerol gelatin (Merck). Pictures were taken using an Olympus BX63 microscope with an Olympus DP73 camera.

**Table 1:** Antibodies used for immunocytochemical and immunohistochemical staining

Marker	Clone	Species	Manufacturer/Order number	Dilution	Antigen retrieval
CD31*	EN4	Mouse	Monosan/MON6002-1	1:300	
CD31#	Polyclonal	Rabbit	Abcam/ab28364	1:100	pH 9
CD34	QBEnd-10	Mouse	Dako/M7165	1:500	pH 6
Vimentin	V9	Mouse	Dako/M0725	1:500*/1:1500^#^	pH 6
vWF	Polyclonal	Rabbit	Sigma/F3520	1:1000*/1:3000^#^	pH 6
SMA	1A4	Mouse	Dako/M0851	1:2500	pH 6
CD45	2B11 + PD7/26	Mouse	Dako/M0701	1:100	pH 6
CK wide	Polyclonal	Rabbit	Abcam/Ab9377	1:200	Pepsin, 250 U/ml

The specific CD31 antibodies used for immunocytochemistry and immunohistochemistry are marked with * and #, respectively. Dilutions used for immunocytochemistry* and immunohistochemistry^#^; antigen retrieval was performed for paraffin embedded tissue as indicated

To confirm endothelial cell identity, surface marker expression of the isolated cells was detected via immunocytochemical staining. To this end, 1 × 10^5^ cells per 1.7 cm^2^ chamber were grown on gelatin pre-coated glass chamber slides (Thermo Fisher Scientific) for 48 h. After washing with HBSS and drying, slides were stored at −20 °C until further processing. Then, slides were fixed with ice-cold acetone (Merck) for 10 min, air dried and rehydrated in TBS. Immunocytochemistry was performed with primary antibodies as indicated in Table 1 using the UltraVision Large Volume Detection System HRP Polymer Kit as described for tissue above, except for TBS usage instead of TBS-T.

**Table 2:** Surface marker antibodies and respective isotype controls used for flow cytometric analysis

Marker	Label	Clone	Manufacturer/Order number	Volume (µl)
Panel 1				
CD14	FITC	TUK4	Miltenyi Biotec/130–080-701	2
CD45	PE	HI30	BD Pharmingen/555483	5
CD133/1	APC	AC133	Miltenyi Biotec/130–090-826	10
CD34	PE-Cy7	581	Beckman Coulter/A21691	4
Panel 2				
CD31	FITC	WM59	BD Pharmingen/560984	5
CD146	PE	P1H12	BD Pharmingen/561013	4
CD90	APC	5E10	BD Pharmingen/561971	0.1
CD309	PE-Cy7	7D4-6	BioLegend/359912	5
CD144	BV421	55-7H1	BD Pharmingen/565670	3
Isotype controls				
FITC		MOPC-21	BD Pharmingen/555748	
PE		MOPC-21	BD Pharmingen/556027	
APC		MOPC-21	BD Pharmingen/555751	
PE-Cy7		679.1Mc7	Beckman Coulter/737662	
BV421		X40	BD Pharmingen/562438	

All antibodies originate from mouse species. Indicated volumes were added to 100 µl of cell suspension

### Phenotypic characterization of placental primary cells via flow cytometry

Flow cytometric analysis was performed as previously described (Leopold et al. 2019) with some minor adaptions. Antibodies are given in Table 2 . Cells were cultured to sub-confluence in a 75 cm^2^ culture flask at 12% O_2_. Detached cells were directly stained after blocking and resuspended afterwards in staining buffer (2 ml for washing, 200 µl before measuring). The viability dye 7-AAD (BD Pharmingen/559925) was added 5 min before measurement to gate on live cells. Compensation was performed by analyzing single color stained OneComp eBeads™ (Thermo Fisher Scientific) that were stained and measured in the same way as the cells. To compensate for 7-AAD, fpAEC were used. Analysis was performed using FlowJo™ version 10 software (FlowJo LLC, OR, USA).

**Table 3:** Percentage of cells positive for indicated surface markers as determined by flow cytometry analysis

Marker	FTUEC	fpAEC	ECFC	HUVEC
CD14	0.04	0.07	0.00	0.08
CD45	0.03	0.08	0.30	0.11
CD90	1.35	0.76	1.49	1.53
CD133	0.00	0.00	0.01	0.09
CD34	98.30	9.81	6.56	16.40
CD31	99.9	99.8	99.9	100.0
CD144	99.8	99.8	99.9	99.8
CD146	100.0	100.0	100.0	100.0
CD309	100.0	100.0	100.0	99.9

### Analysis of proliferative capacity

Proliferation was analyzed using a Click-iT Plus EdU Imaging Kit (Life Technologies, CA, USA), which allows measurement of the incorporation of the modified thymidine analog EdU (5-ethynyl-2′-deoxyuridine) during DNA replication and subsequent click reaction-based detection. Cells (1.5 × 10^4^ cells/well) were seeded on gelatin-pre-coated glass chamber slides and incubated at 12% O_2_, 5% CO_2_ at 37 °C in a humidified incubator. Experiments were performed in quadruplicate, and the proliferation rate was measured at 0 and 24 h. The 0 h time point was defined as the point at which cells attached to the slide (approx. 4 h after seeding). For both time points, 500 µl of the culture medium was replaced with 2× EdU working solution (20 µM) diluted in culture medium to a final concentration of 10 µM. After incubation for 1 h, medium was removed, wells washed with 1 ml HBSS, dried, and stored at −20 °C until further processing. Then, cells were fixed with ice-cold acetone and further processed according to the manufacturer’s protocol to enable the Click-iT reaction and the staining of the nucleus with Hoechst^®^ 33342. For analysis, 15 equally distributed pictures of each chamber from all slides and time points were taken with an Olympus BX63 microscope equipped with an Olympus DP73 camera. Images were analyzed automatically with the free software CellProfiler version 4.1.3 (McQuin et al. 2018).

### Analysis of absolute cell numbers

A total of 1.2 × 10^5^ cells/well were seeded in 2 ml culture medium in 6-well culture plates pre-coated with gelatin and incubated at 12% O_2_, 5% CO_2_ at 37 °C in a humidified incubator. After 24, 48 and 72 h, cell numbers were determined using a CASY TT Analyzer System (OLS, Omni Life Science, Bremen, Germany). For that purpose, medium was aspirated, cells washed with HBSS, and each well incubated with 500 µl TrypLE™ at 37 °C for 5 min. One milliliter of culture medium was added to the detached cells and transferred into 2 ml tubes (Eppendorf, Hamburg, Germany). 100 µl of the cell suspension was added to 9.9 ml CASYton (CASY electrolyte solution) and counted in a triple measurement mode. Normalization and evaluation cursors were set to 7.88 and 12 µm for FTUEC and fpAEC and 7.88 and 11 µm for HUVEC and ECFC, to discriminate between cell debris, dead and living cells. Experiments were performed in triplicate.

## Results

### Endothelial cell isolation by flow-through digestion of first trimester umbilical cord vessel

For enzymatic flow-through digestion of the umbilical endothelium of human first trimester placenta, we used the umbilical cord dissected close to the cord insertion (Fig. 1a–c). Initial proof-of-concept experiments showed that cannulation of human first trimester umbilical cord vessels, using micro-catheters designed for parenteral nutrition and drug application in neonatal care, was feasible from gestational week 8 onwards. Moreover, sequential cannulation of all three umbilical cord vessels was possible within a reasonable time frame of 30 min (Fig. 1d–f). Unsuccessful cannulation was detected macroscopically when a puncturing of the vessel walls resulted in a clear penetration of the Wharton's jelly, i.e. the extraembryonic mesoblast-derived connective tissue of the cord. Successful cannulation was confirmed by formalin fixation and paraffin embedding of cannulated umbilical cords with subsequent histological tissue processing. HE staining of cannulated umbilical cord sections showed correct positioning of the catheters inside the cord vessels (Fig. 1g). It is worth mentioning that the cord vessels were completely filled with the catheters (outer diameter of 330 µm), suggesting a certain dilatability of the vessel walls.

**Fig. 2:**
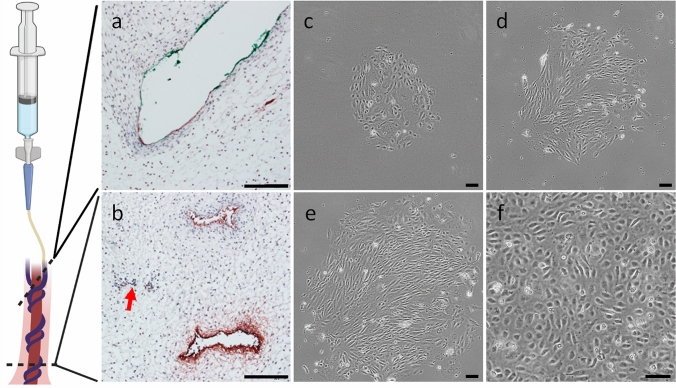
Isolation and culture of first trimester umbilical cord endothelial cells. After enzymatic flow-through digestion of the umbilical cord endothelium, the FFPE cord was subjected to histological examination for vWF staining at the proximal (**a**) and distal (**b**) sides of catheter insertion. At the side proximal to catheter positioning, remnants of tissue marking dye (green) were detected on the endothelial lining (**a**). At the distal side (**b**), distinct vWF staining revealed the presence of endothelia in two cord vessels, whereas the third vessel lacked immunostaining due to depletion of EC (red arrow). After 7 days in culture, FTUEC appeared as small colonies (**c**), which further expanded after 8 (**d**) and 9 (**e**) days in culture. Following first passaging, cells achieved appropriate confluence (**f**). Scale bars represent 100 µm. Figure parts were created with BioRender.com

To confirm the efficiency of enzymatic flow-through digestion of the umbilical cord endothelium, FFPE umbilical cord sections were stained after the isolation procedure for the classical EC marker von Willebrand factor (vWF). In the case of single cannulation, the catheter was dyed with a green tissue marking dye before insertion, enabling identification of the cannulated cord vessel in FFPE umbilical cord sections. At the proximal side of catheter insertion, green staining was detected on the endothelial lining of one vessel, indicating transfer of tissue marking dye from the catheter to the cannulated tissue (Fig. 2a). At the distal segment, distinct vWF staining revealed the presence of two cord vessels, whereas the third vessel lacked immunostaining due to enzymatic depletion of ECs (Fig. 2b).

The initial cultivation of the enzymatic digestion fractions showed a heterogeneous picture in terms of colonies formed by the freshly isolated FTUEC; 13, 9, 5, and 2 colonies grew out after 2, 4, 6, and 8 min of enzymatic digestion, respectively. Hence, the incubation time for enzymatic flow-through digestion may be minimized to 4–6 min. The isolated FTUEC showed proper colony outgrowth with an endothelial-like morphology (Fig. 2c–e). The cells also maintained their polygonal appearance by forming monolayers with classical endothelial cobblestone morphology, also after passaging 10 days after isolation (Fig. 2f).

### Phenotype of FTUEC in vitro and in situ

**Fig. 3:**
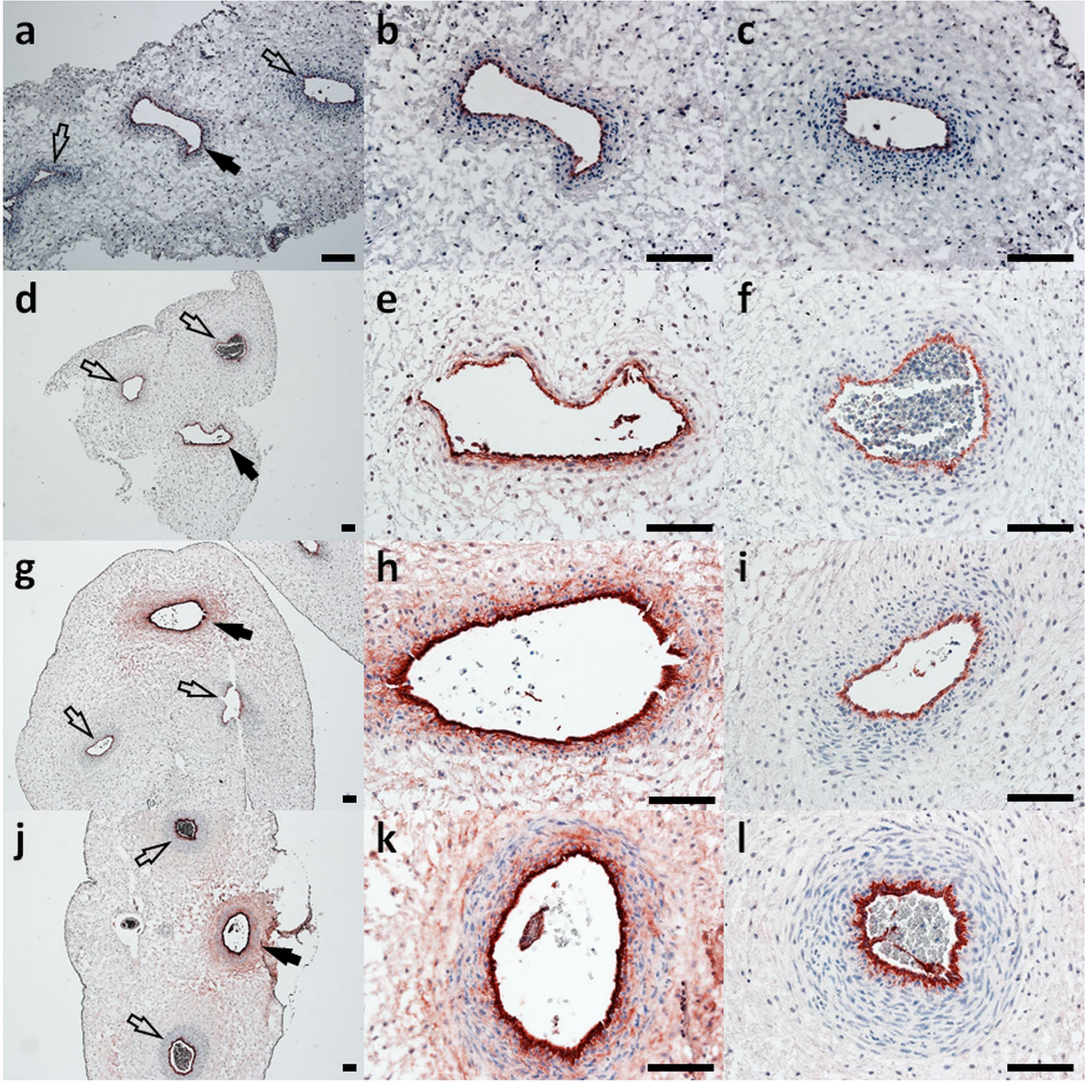
Immunohistochemistry of vWF in human first trimester umbilical cord tissue. Immunohistochemistry for vWF in umbilical cord tissue at gestational age 7+3 (**a**–**c**), 9+5 (**d**–**f**), 10+3 (**g**–**i**), and 11+6 (**j**–**l**) revealed a gestational age-dependent increase in subendothelial and perivascular vWF location in umbilical veins (black arrows, and in higher magnification in **b**, **e**, **h**, and **k**), while vWF staining was confined to the endothelia of umbilical arteries (open arrows, and in higher magnification in **c**, **f**, **i**, and **l**). Scale bars represent 100 µm

In a next step, we analyzed phenotype and purity of FTUEC in vitro and in situ by immunocytochemistry. Interestingly, vWF, a marker for endothelial cells, showed a different staining pattern for the two arteries compared with the vein of human first trimester umbilical cord vessels (Fig. 3). While vWF staining was clearly confined to the arterial endothelia, the staining of the venous endothelium was more intense, but appeared diffuse, including staining of subendothelial areas. The intensity of vWF in umbilical vein subendothelial areas increased with gestational age, suggesting a developmental correlation.

**Fig. 4:**
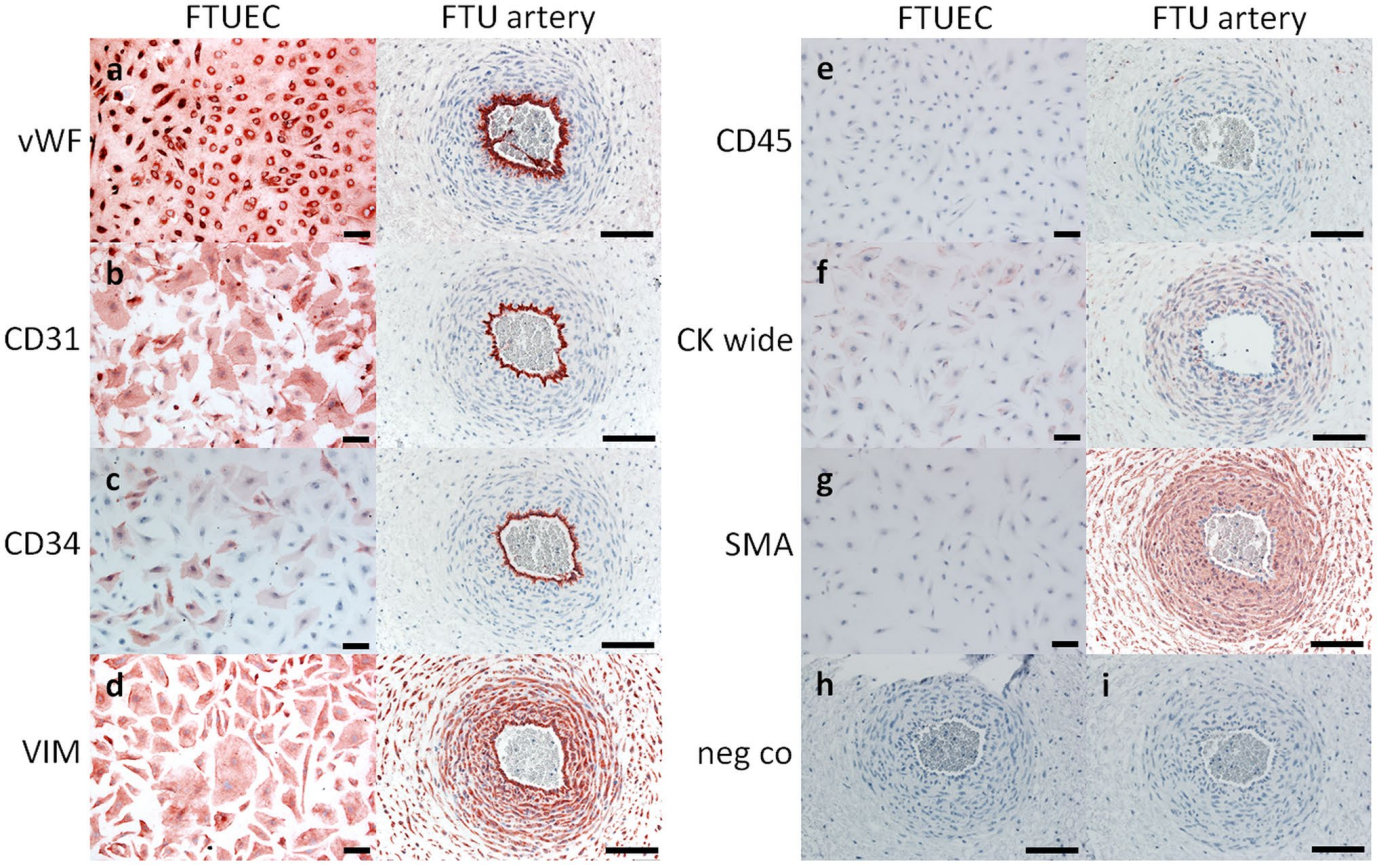
Immunostaining of FTUEC and first trimester umbilical cord tissue**.** Immunocytochemistry for vWF (**a**) and CD31 (**b**) showed strong staining of FTUEC and in situ umbilical cord arteries. CD34 (**c**) was moderately stained in a proportion of FTUEC, while the endothelium of umbilical arteries was distinctly stained. FTUEC as well as umbilical endothelium was also stained for vimentin (VIM) (**d**), as was the tunica media and the surrounding Wharton's jelly in umbilical cords. Staining for CD45 (**e**) and pan cytokeratin (CK wide, **f**) gave no signals either in cells or in umbilical cord sections. Staining for smooth muscle actin (SMA, **g**) was negative in FTUEC, and predominantly stained the tunica media of umbilical blood vessels. Negative controls for mouse (**h**) and rabbit (**i**) IgG showed no staining. Scale bars represent 100 µm

Both endothelial cell markers, i.e. vWF and CD31, were markedly stained in FTUEC and the endothelium of umbilical cord vessels (Fig. 4a, b). Consistent with previous observations in HUVEC (Gogia et al. 2017), FTUEC showed a perinuclear localization of vWF (Fig. 4a). CD34, a marker for mesenchymal stem cells, endothelial progenitor cells, and some endothelial cell populations, was moderately stained in culture, whilst the endothelium of umbilical blood vessels was distinctly stained (Fig. 4c), confirming previous observations (Blaschitz et al. 2011). To investigate whether the expression of the progenitor marker CD34 diminishes throughout culture, FTUEC were propagated until passage 10 and subjected to immunocytochemistry. Cells remained CD34-positive until passage 10 (not shown). Immunocytochemistry for vimentin, a marker for mesenchymal and endothelial cells, confirmed the presence of the intermediate filament in FTUEC as well as the umbilical endothelium, the tunica media, and the surrounding Wharton's jelly (Fig. 4d). While CD45, a marker for hematopoietic progenitor cells and leukocytes, was not detected in either isolated FTUEC or the entire umbilical cord sections (Fig. 4e), staining for pan cytokeratin (CK wide) was only detected in the amniotic epithelium (Online Resource 1). Absence of cytokeratin staining in FTUEC monolayers proved that isolated cells were devoid of possible contaminating amniotic epithelial cells (Fig. 4f). Likewise, staining for smooth muscle actin (SMA) was negative in FTUEC and in situ umbilical cord endothelium (Fig. 4g). As expected, SMA staining was predominantly detected in the tunica media of umbilical blood vessels and to a minor extent in the perivascular Wharton’s jelly, where SMA-positive myofibroblasts may participate in turgor regulation of the cord, preventing compression of the umbilical vessels and counteracting bending or kinking of the cord.

### Phenotype of FTUEC versus primary fetal EC from term pregnancy

**Fig. 5:**
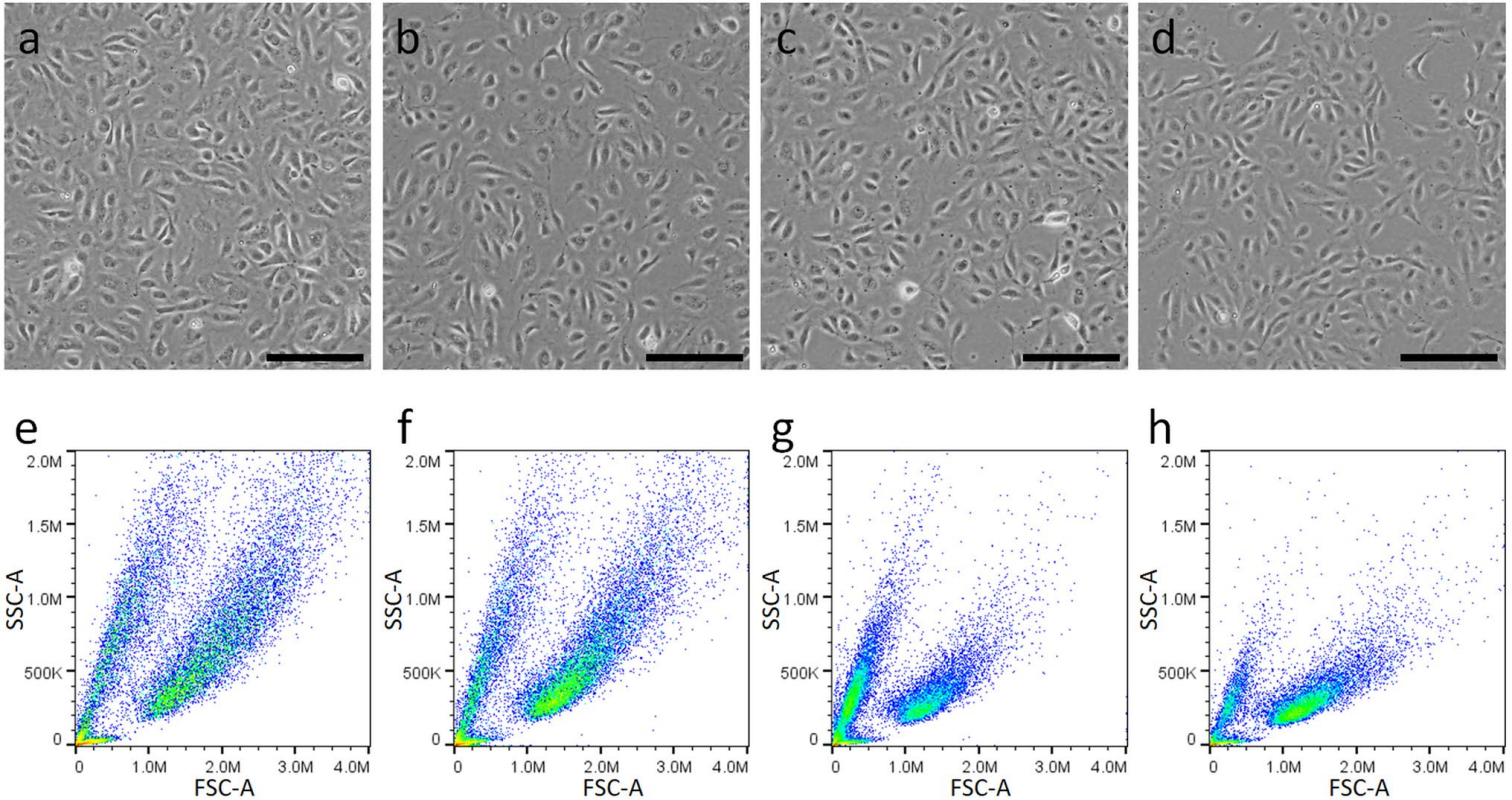
Cellular morphology and size distribution of FTUEC and term pregnancy endothelial cells**.** FTUEC (**a**, **e**), fpAEC (**b**, **f**), ECFC (**c**, **g**), and HUVEC (**d**, **h**) were grown to 80–90% confluence (**a**–**d**) and subjected to flow cytometric analysis without staining, separated according to size and granularity (**e**–**h**). Scale bars represent 200 µm

FTUEC and EC from term pregnancy were immunophenotyped by flow cytometry. Although the microscopic appearance of cultured ECs was similar, with typical endothelial cobblestone morphology in a monolayer (Fig. 5a–d), size and granularity distribution differed. This became obvious in flow cytometric analysis looking at the plotted side scatter area (SSC-A) as hallmark for cell granularity/complexity versus forward scatter area (FSC-A) indicating cell size (Fig. 5e–h). While FTUEC (Fig. 5e) were similar in size but slightly more extended to high granularity compared to fpAEC (Fig. 5f), ECFC (Fig. 5g) and HUVEC (Fig. 5h) were similar in both size and granularity. Based on these variations, the axis scales for each EC type needed to be adapted in order to investigate the proportion of surface marker expression. After gating on the respective cell population, cell doublets and dead cells were excluded. For that purpose, cells were stained with the viability dye 7-AAD, resulting in a positive signal in dead cells, where the dye enters through disrupted membranes. Within live cells, we then compared particular marker expression of FTUEC to the other investigated EC types by plotting single parameters and gating depending on the respective isotype control (Online Resource 2). All investigated cell types were negative for the monocyte/macrophage marker CD14, for CD45, and for the stem cells marker CD133. In all cell types, a tiny population positive for CD90 (0.76–1.53%), a marker for stem cells/fibroblasts, was observed. All cell types showed positive expression of the EC markers CD31, CD144, CD146, and CD309. Intriguingly, almost all FTUEC showed positive staining for the progenitor marker CD34 (98.30%), whereas only a small population was positive in EC isolated at term of pregnancy (6.56–16.40%) (Table 3). Again, we analyzed the effect of cultivation on CD34 expression of FTUEC and subjected cells between passage 7 and 10 to flow cytometry. In parallel with the results obtained by immunocytochemistry, which revealed persistent CD34 expression, FTUEC remained CD34-positive (> 90%) from passage 3 up to passage 10 (not shown).

### Proliferation of FTUEC versus EC from term pregnancy

**Fig. 6:**
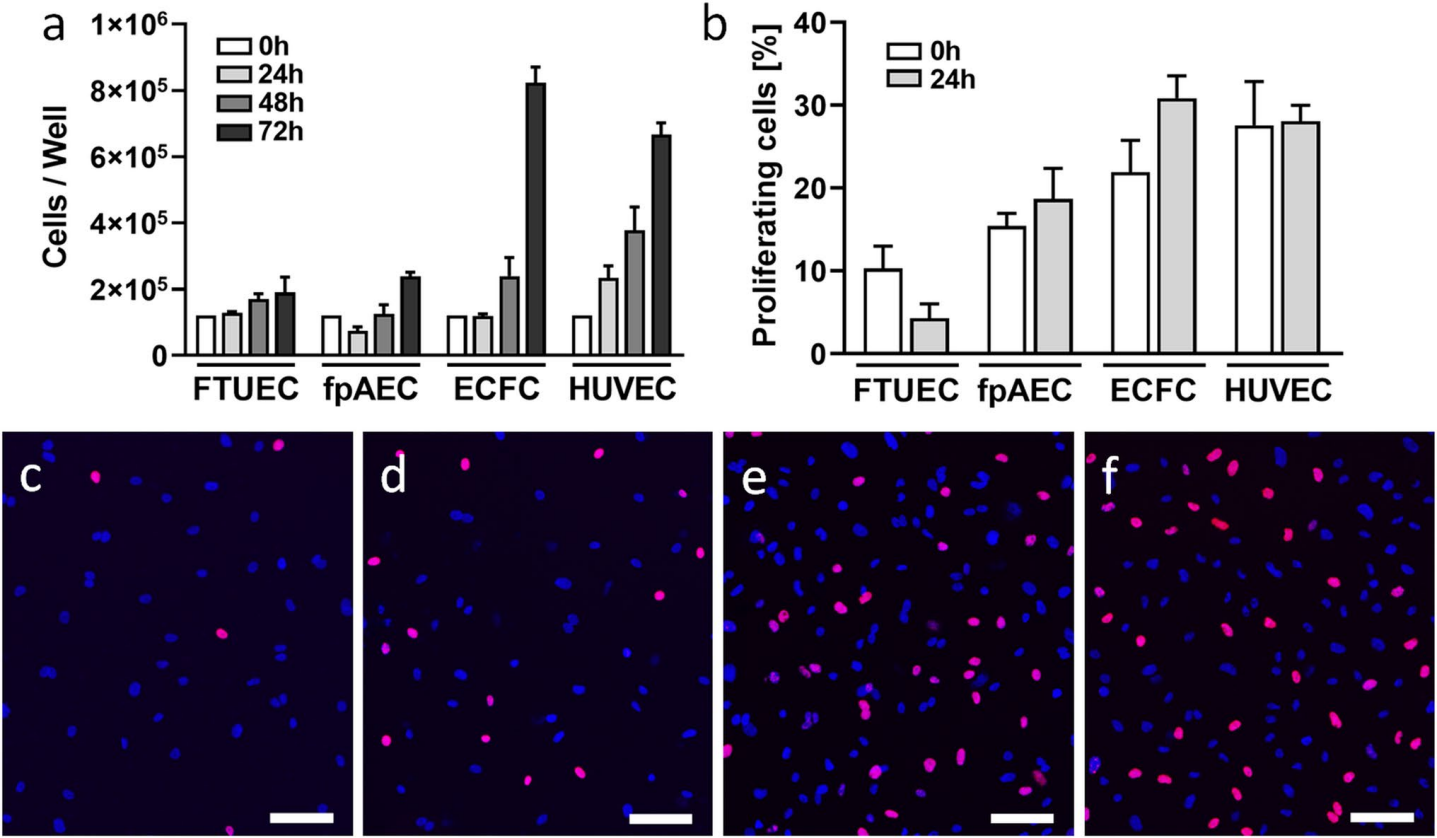
Proliferation of FTUEC and term pregnancy endothelial cells**.** Absolute cell numbers of placenta- and cord blood-derived EC were determined by electric field multichannel cell counting at indicated time points (**a**). Cell proliferation, based on EdU incorporation, was analyzed by software-based image analysis at experimental start, i.e. after adherence of cells at 0 h, and 24 h of culture (**b**). Representative images are shown for FTUEC (**c**), fpAEC (**d**), ECFC (**e**), and HUVEC (**f**) after 24 h of culture. Blue Hoechst^®^ 33342 dye marks all cells; the purple stain is specific for proliferating cells. Scale bar represents 100 µm

Finally, the proliferative capacity was compared between FTUEC and EC from term pregnancy. For this purpose, cell numbers of FTUEC, fpAEC, ECFC, and HUVEC were analyzed every 24 h over a period of 3 days using an electric field multichannel cell counting system (CASY) (Fig. 6a). After 24 h in culture, cell numbers were similar between analyzed cells, except HUVEC, which showed a steep linear increase right from the beginning. Cell numbers of FTUEC gently increased over time, as did the numbers of fpAEC. However, analysis of ECFC suggested an almost exponential increase in cell numbers, which reached the highest number of all analyzed cell types after 3 days. Since differences in cell numbers may be in part explained by different proliferation rates, FTUEC and EC from term placenta were subjected to proliferation assay, using EdU incorporation as a measure of DNA synthesis. After adherence of cells, FTUEC and fpAEC showed the lowest percentage of proliferating cells, which in FTUEC declined even further after 24 h (Fig. 6b and c). While the percentage of proliferating cells remained almost constant in fpAEC (Fig. 6b and d) and HUVEC (Fig. 6b and f) after 24 h, values in ECFC (Fig. 6b and e) further increased, explaining the exponential increase in cell numbers observed by cell counting.

## Discussion

We have developed a new method to isolate pure and expandable EC from human first trimester umbilical cord vessels. Similar to EC isolated from feto-placental tissues after delivery at term of pregnancy, FTUEC display an endothelial phenotype (positive expression of CD31 [PECAM1], CD144 [VE-cadherin], CD146 [endothelial-associated antigen MCAM], and CD309 [VEGF receptor KDR]), with a lack of the hematopoietic progenitor markers CD45 and CD133 (Timmermans et al. 2009) or CD14, which is expressed on a progenitor cell population that differentiate in EC with low proliferative capacity (Krenning et al. 2009). However, in contrast to EC from term pregnancy, FTUEC were highly positive for the hematopoietic progenitor cell antigen CD34. CD34 is a marker for very functional (Yang et al. 2011) and angiogenic (Siemerink et al. 2012), juvenile (Ferreras et al. 2015) endothelial subpopulations. This strongly suggests that FTUEC represent an early endothelial phenotype, capable of promoting the expansion and differentiation of the placental and fetal vasculature in the first trimester of pregnancy. Any impairment of these highly active cells by negative external influences, such as maternal pathologies, may have permanent effects on the fetal and placental vasculature.

FTUEC show a perinuclear localization of vWF, a multimeric glycoprotein that contributes to platelet adhesion and hemostatic plug formation at sites of vascular injury. The perinuclear localization of vWF in FUTEC is in good agreement with previous observations in EC, showing vWF in endothelial cell-specific and highly organized storage vesicles, referred to as Weibel-Palade bodies (Romani de Wit et al. 2003). To the best of our knowledge, we are the first to show a differential staining pattern for vWF in human first trimester umbilical cord vessels. vWF was clearly confined to the arterial endothelium, whereas in the vein, subendothelial areas are positive as well. Whether active basolateral secretion processes from the ECs or just leakage through the umbilical venous endothelium causes the observed differential staining pattern remains to be clarified. However, the fact that previous real-time analyses of electrical impedance showed a better barrier function in human feto-placental venous than arterial EC (Cvitic et al. 2018) rather excludes the scenario of increased vWF leakage from the vessel into the umbilical venous subendothelial area. Hence, active secretion of vWF from the basolateral side of ECs to the subendothelial matrix, as described for HUVEC (Lopes da Silva and Cutler 2016), seems very likely. Constitutive secretion of vWF into the subendothelial matrix has been suggested to play a role in platelet recruitment following exposure by injury (Patella and Cutler 2020). Thus, it is tempting to speculate that the umbilical vein is more vulnerable to thrombotic events, where subendothelial vWF may play a significant role in hemostasis after partial ruptures at sites of cord compression.

In comparison to the endothelial cell models from term pregnancy, FTUEC revealed lower proliferation. Importantly, we observed that FTUEC are very sensitive towards seeding density, suggesting a link to cell growth and proliferation. Due to their relatively large size, cultured FTUEC monolayers reached confluence earlier than term ECs, which consequently may have led to contact inhibition of cell proliferation, in which VE-cadherin has been suggested as a key regulator (Wallez and Huber 2008; Dejana and Giampietro 2012). Induction of contact inhibition guarantees that cells stop proliferation once they have reached confluence. The slower proliferation rate found in FTUEC parallels their CD34^+^ phenotype, which has been described as a tip-cell characteristic (Siemerink et al. 2012). In fact, tip cells exhibit lower proliferation compared to stalk cells, which provide the cell pool for the growing vessel (Sainson et al. 2005).

This is the first report describing a method for isolation of pure and expandable placental/umbilical cord EC from the first trimester of pregnancy. As a clear advantage of our approach, we see the reduced cellular stress due to the fast procedure and the lack of positive or negative selection steps. Another advantage of our method is that, similar to third trimester umbilical EC, arterial and venous cells can be isolated, even from the same cord. Finally, in comparison to antibody-based cell separation strategies, isolation by enzymatic perfusion is less expensive. In fact, a protocol for isolation of first trimester placental EC has been developed previously using enzymatic and mechanical forces to obtain a placental cell suspension followed by positive immunomagnetic selection of EC (Charolidi et al. 2019). The obtained cells showed an endothelial phenotype (vWF^+^, CD31^+^) and formed colonies. However, no information can be given regarding purity, passaging, and/or long-term culture, as the cells were not tested for contamination and were used within 24 h after isolation. A limitation of our approach may be the restricted availability of intact first trimester umbilical cords. Even if access to first trimester placental tissue is warranted, surgical termination of pregnancy may damage the tissue, resulting in too short cord fragments not suitable for the flow-through isolation procedure.

We see potential applications and fields of use for FTUEC in research into the development of the fetal and placental vasculature. Studying the plasticity of placenta-derived ECs and their potential pheno-/genotypic change over gestation represents an intriguing future topic in the field, rendering FTUEC a valuable tool. Whether our current methodological approach could be extended to ex vivo dual-side perfusion of first trimester placenta via the umbilical vessels, again, depends on tissue integrity, and not least on the technical experience of the experimenter. However, the effect of various substances, metabolites, and hormones on the function of the endothelium of the first trimester can be studied in vitro in FTUEC monolayers as well. Finally, the impact of exposure to maternal derangements such as cigarette smoking, obesity, hypertension, and hormonal disorders on viability, function, gene expression, and programming of the early endothelium can be investigated. Hence, FTUEC represent a novel tool to gain insight into endothelial function and dysfunction in early development.

## Supplementary Information

Below is the link to the electronic supplementary material.

Supplementary file1 (PDF 568 KB)

Supplementary file2 (PDF 1017 KB)

## Data Availability

All data generated or analyzed during this study are included in this published article and its supplementary information files.
