# Supplementary material for: Flow-through isolation of human first trimester umbilical cord endothelial cells
Source: Histochem Cell Biol. 2021 Jun 24;156(4):363–75. doi: 10.1007/s00418-021-02007-7 (PMC8550006; doi:10.1007/s00418-021-02007-7)
Supplement: Supplementary file 1 — Supplementary file1 (PDF 568 KB) [file 418_2021_2007_MOESM1_ESM.pdf]

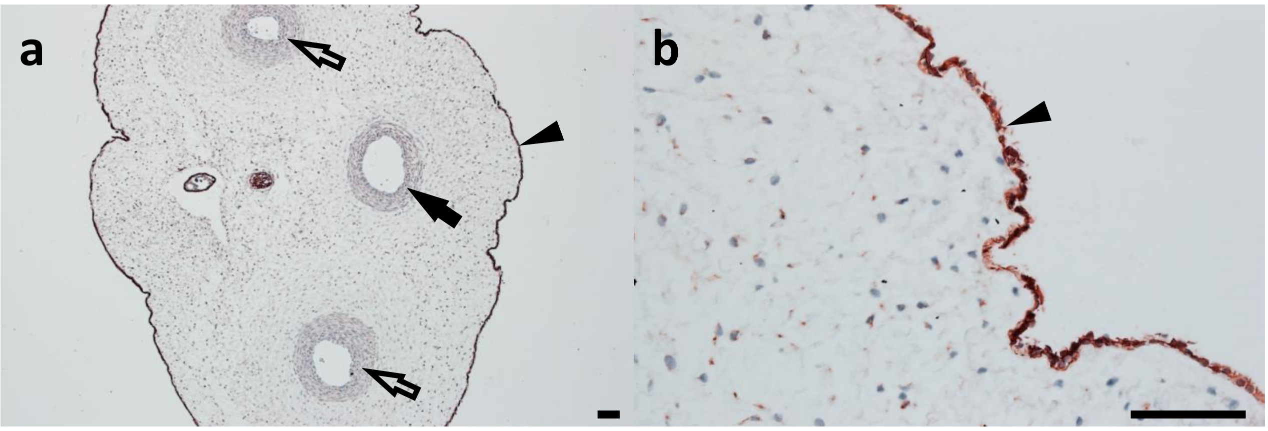


**Online Resource Figure 1**

*Immunohistochemistry of human first trimester umbilical cord for pan-cytokeratin*

Staining of first trimester umbilical cord for pan-cytokeratin was detected in the amniotic epithelium (arrowheads in **a** and **b**), whereas endothelia of umbilical arteries (open arrows in **a**) and the vein (closed arrows in **a**) were negative. Note the allantoic remnants located centrally between the two umbilical arteries (**a**), showing positively stained epithelial cells. Scale bars represent 100 µm.
