# Supplementary material for: Flow-through isolation of human first trimester umbilical cord endothelial cells
Source: Histochem Cell Biol. 2021 Jun 24;156(4):363–75. doi: 10.1007/s00418-021-02007-7 (PMC8550006; doi:10.1007/s00418-021-02007-7)
Supplement: Supplementary file 2 — Supplementary file2 (PDF 1017 KB) [file 418_2021_2007_MOESM2_ESM.pdf]

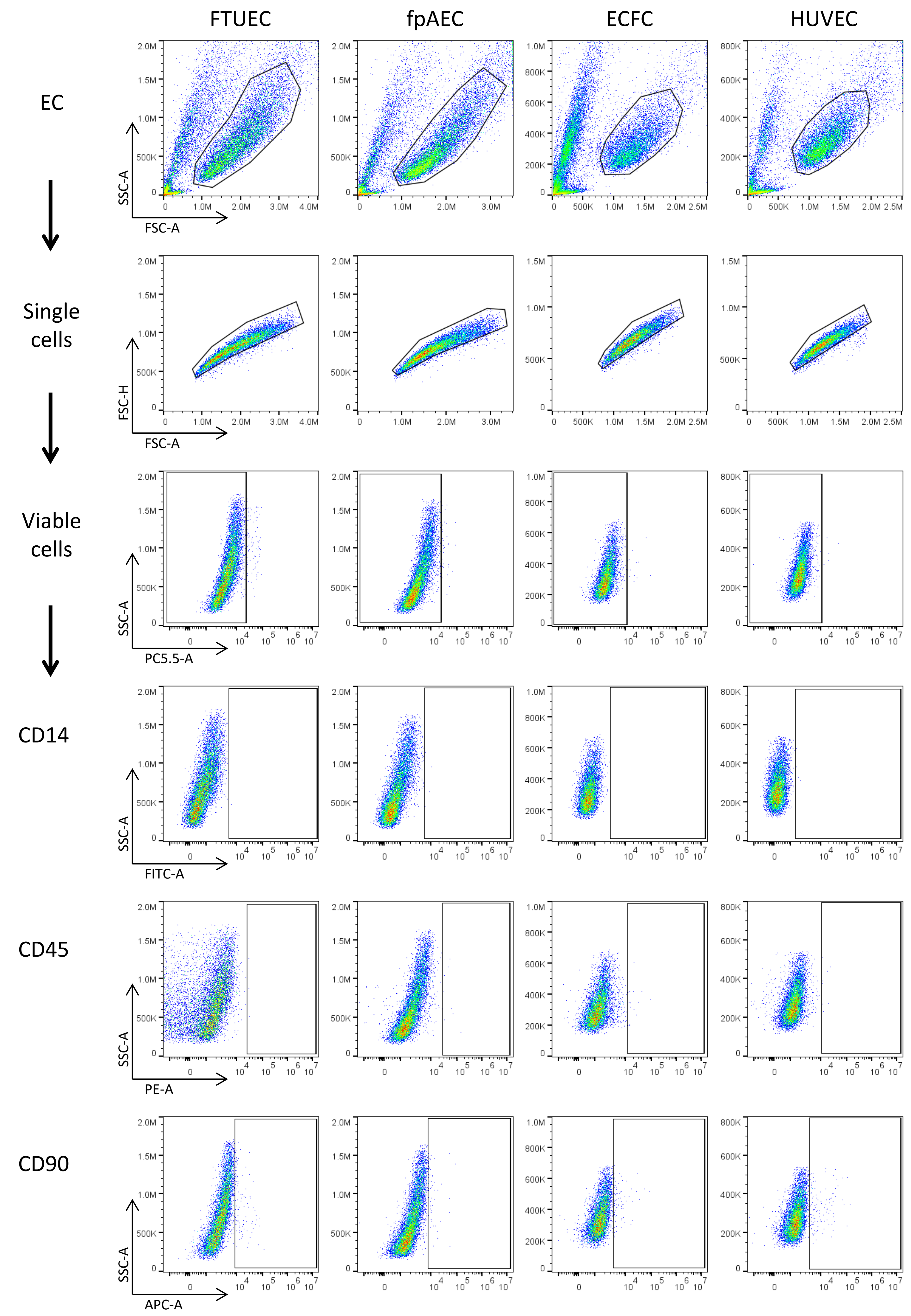
FTUEC
fpAEC
ECFC
HUVEC
EC
Single cells
Viable cells
CD14
CD45
CD90
SSC-A
FSC-A
FSC-H
PC5.5-A
FITC-A
PE-A
APC-A

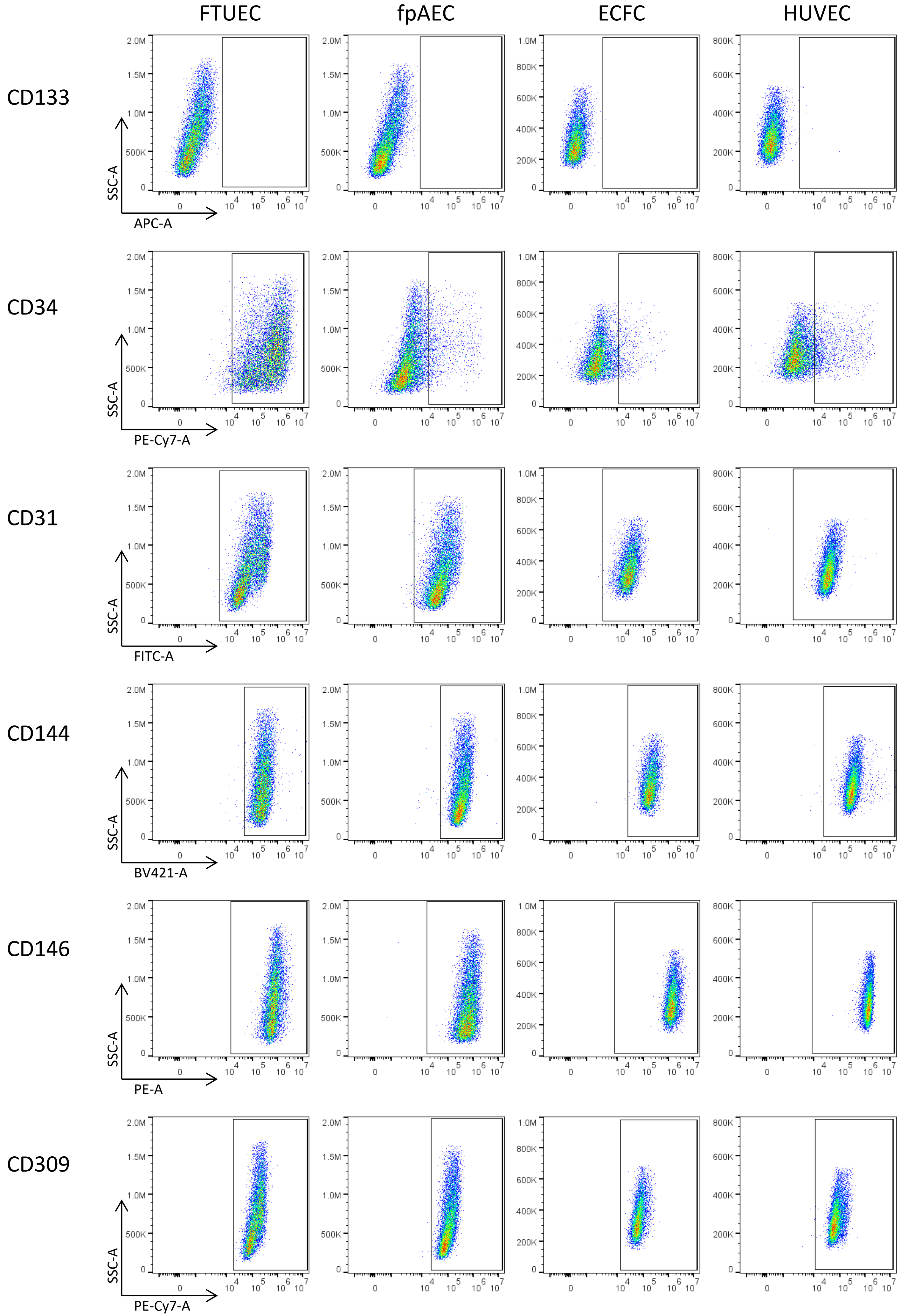
FTUEC
fpAEC
ECFC
HUVEC
CD133
CD34
CD31
CD144
CD146
CD309
SSC-A
APC-A
PE-Cy7-A
FITC-A
BV421-A
PE-A
PE-Cy7-A

Online Resource Figure 2

Gating strategy of flow cytometry analysis

Isolated cell types were stained with established marker panels to identify cell phenotype and purity. After gating on the cell population as well as single and viable cells, single marker expression was analyzed by plotting SSC-A vs the respective fluorochrome with gating depending on the corresponding isotype control.
